# *α*-C(sp^3^)-H Arylation of Cyclic Carbonyl Compounds

**DOI:** 10.1007/s13659-021-00312-1

**Published:** 2021-06-07

**Authors:** Mei Wang, Wei Wang, Dashan Li, Wen-Jing Wang, Rui Zhan, Li-Dong Shao

**Affiliations:** 1 Yunnan Key Laboratory of Southern Medicinal Utilization, School of Chinese Materia Medica, Yunnan University of Chinese Medicine, Kunming, 650050 China; 2 School of Chemistry and Chemical Engineering, Yunnan Normal University, Kunming, 650050 China

**Keywords:** C-H functionalization, *α*-C(sp^3^)-H arylation, Cyclic carbonyl compounds

## Abstract

*α*-C(sp^3^)-H arylation is an important type of C-H functionalization. Various biologically significant natural products, chemical intermediates, and drugs have been effectively prepared via C-H functionalization. Cyclic carbonyl compounds comprise of cyclic ketones, enones, lactones, and lactams. The *α*-C(sp^3^)-H arylation of these compounds have been exhibited high efficiency in forming C(sp^3^)-C(sp^2^) bonds, played a crucial role in organic synthesis, and attracted majority of interests from organic and medicinal communities. This review focused on the most significant advances including methods, mechanism, and applications in total synthesis of natural products in the field of *α*-C(sp^3^)-H arylations of cyclic carbonyl compounds in recent years.

## Introduction

**Scheme 1:**
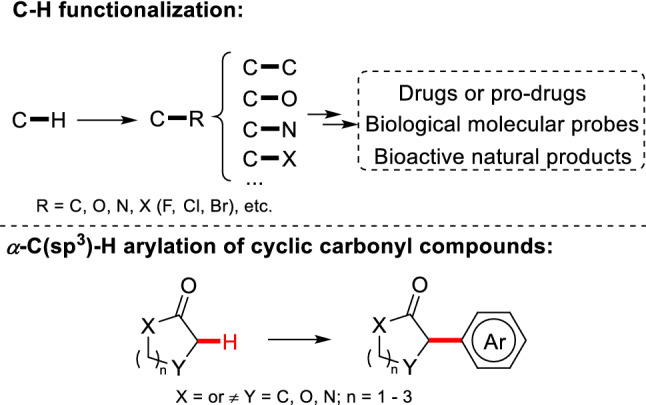
C-H functionalization and *α*-C(sp^3^)-H arylation of cyclic carbonyl compounds

C-H functionalization is the most powerful method that directly transforms C-H bond to C-R bond (R is any atom except H, such as C, O, N, etc.) (Scheme 1). Since C-H bonds are common in organic compounds, C-H functionalization has an extremely broad space of application. Through direct C-H functionalization, the functionalized substrate [1–7], drug intermediates [8–10], and bioactive natural products with complex structures [11–15] can be successfully synthesized in a highly effective manner. Therefore, it has been regarded as an "eternal theme" in organic chemistry [16, 17]. As an important type of C-H functionalization, the *α*-C(sp^3^)-H arylations of cyclic carbonyl compounds (cyclic ketones, enones, lactones and lactams) are affected by ring tension, steric hindrance, and electronic effect, which have been considered to be a tough issue [18, 19]. Considering the important application of the *α*-C(sp^3^)-H arylations of cyclic carbonyl compounds in organic chemistry, medicinal chemistry, and drug development process. Previous reviews summarized the progress in Pd-catalyzed *α*-C(sp^3^)-H arylations of carbonyl compounds [20–22], the applications of these reactions in the total synthesis of natural products [23], and the enantioselective approaches [24]. This review mainly focused on the conditions, substrates ranges and the application of *α*-C(sp^3^)-H arylation of cyclic carbonyl compounds in recent years.

## Transition-Metal Catalyzed ***α***-C(sp^3^)-H Arylation of Cyclic Carbonyl Compounds

### Palladium-Catalyzed ***α***-C(sp^3^)-H Arylation of Cyclic Carbonyl Compounds

**Scheme 2:**
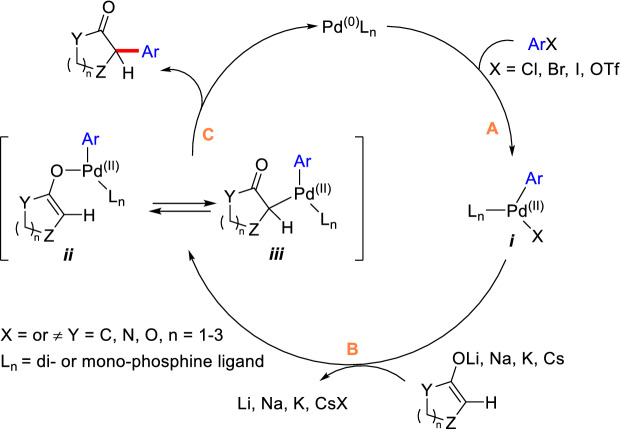
Proposed mechanism for the Pd-catalyzed *α*-C(sp^3^)-H arylation of cyclic carbonyl compounds [25]

Palladium is the most widely used catalyst in *α*-C(sp^3^)-H arylation of cyclic carbonyl compounds, featuring with small amount of catalyst loading and simple operation. The mechanism of well-established Pd-catalyzed *α*-C(sp^3^)-H arylation has been proposed as shown in Scheme 2. The generally catalytic cycle comprises of three paths (A-C). Oxidative addition of Pd(0) with ArX (X = Cl, Br, I, and OTf) forms complex ***i*** (path A). Transmetallation of intermediate ***i ***with enolates (Li, Na, K, Cs, etc.) provide active enol-palladium complexes ***ii*** which can isomerize to ***iii*** (path B) [19, 25]. The organopalladium intermediate ***iii*** finally converts to *α*-aryl compound through reductive elimination (path C).

#### Palladium-Catalyzed Intramolecular ***α***-C(sp^3^)-H Arylations of Cyclic Carbonyl Compounds

**Scheme 3:**
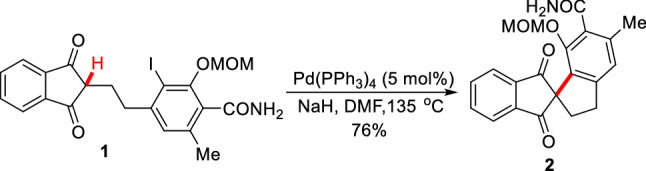
Intramolecular *α*-C(sp^3^)-H arylation of 1,3-cyclopentadienone **1**

**Scheme 4:**
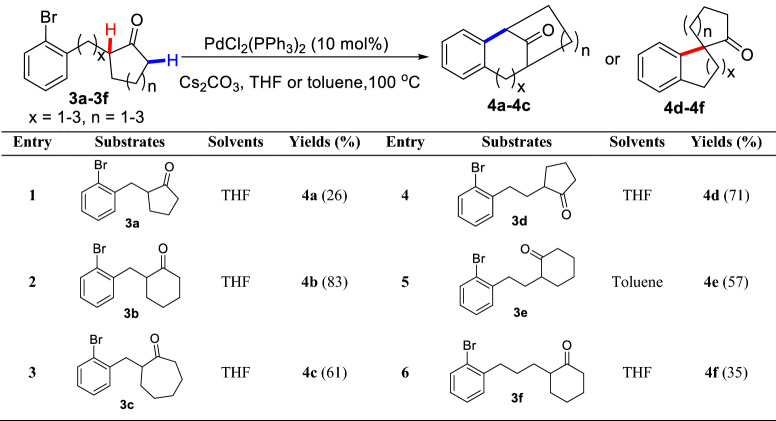
Intramolecular *α*-C(sp^3^)-H arylation of *α*-bromoaryl substituted cyclic ketone **3a-3f**

**Scheme 5:**
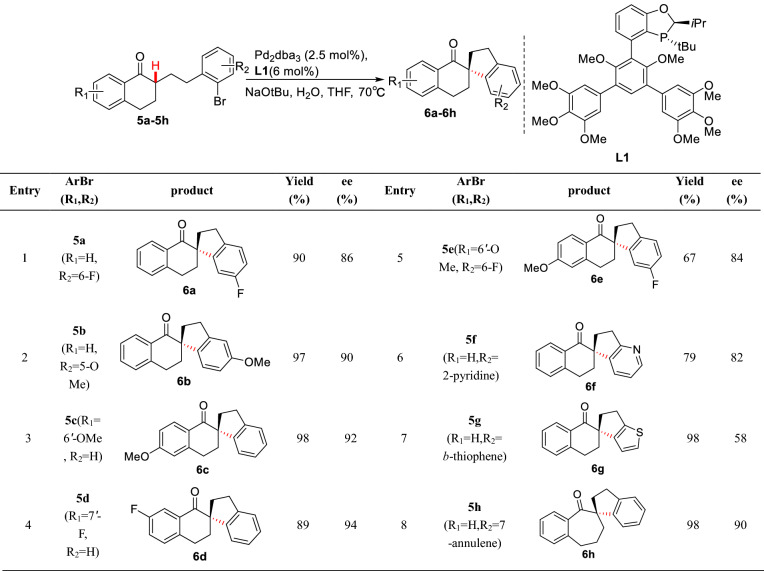
Spiro-ketones formed via intramolecular enantioselective *α*-arylation

**Scheme 6:**
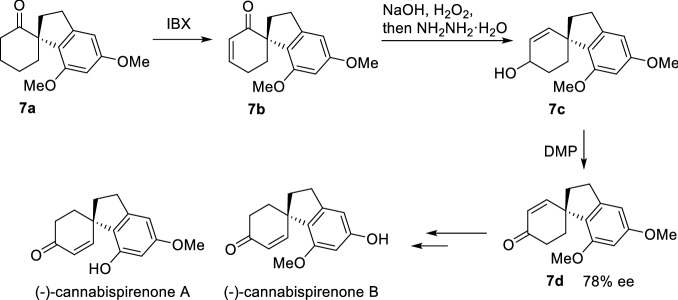
Formal syntheses of (−)-cannabispirenones A and B

In 1988, Ciufolini et al. reported a Pd-catalyzed intramolecular *α*-arylation of 1,3-cyclopentadienone **1**, and suggested that forming the "soft" enolate by NaH could significantly improve the yield of **2** (Scheme 3)[26]. Later, Natsume et al. reported a similar Pd-catalyzed *α*-arylation of cyclic ketone (**3a-3f**), and found that bridge rings (**4a**-**4c**) or spiro rings (**4d-4f**) could be prepared by controlling the lengths of *α* side chains (Scheme 4) [27]. With the strategy, a gram-scale total synthesis of (-)-huperzine A was achieved by Herzon et al. [28]. To achieve an enantioselective intramolecular arylation of **3d**-based analogue, the coordinated catalyst JosiPhos/Pd(OAc)_2_ was established by Sasai et al. [29]. Recently, Tang et al. described an asymmetric intramolecular *α*-arylation that provided good to excellent enantioselectivities for a wide range of spirocyclic structures (**5a-5h**) by developing an effective *P*-chiral monophosphorus ligand **L1** (Scheme 5) [30]. The method also enabled the formal syntheses of (-)-cannabispirenones A and B (Scheme 6) [30].

**Scheme 7:**
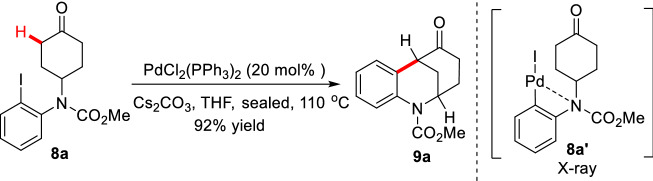
Intramolecular *α*-arylation of functionalized cyclohexanone **8a**

**Scheme 8:**
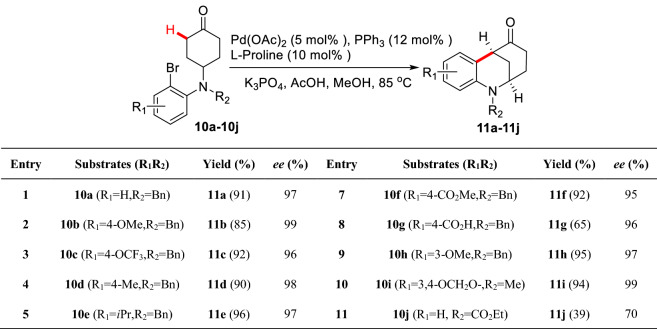
Desymmetric enantioselective *α*-C(sp^3^)-H arylations of functionalized cyclohexanones

**Scheme 9:**
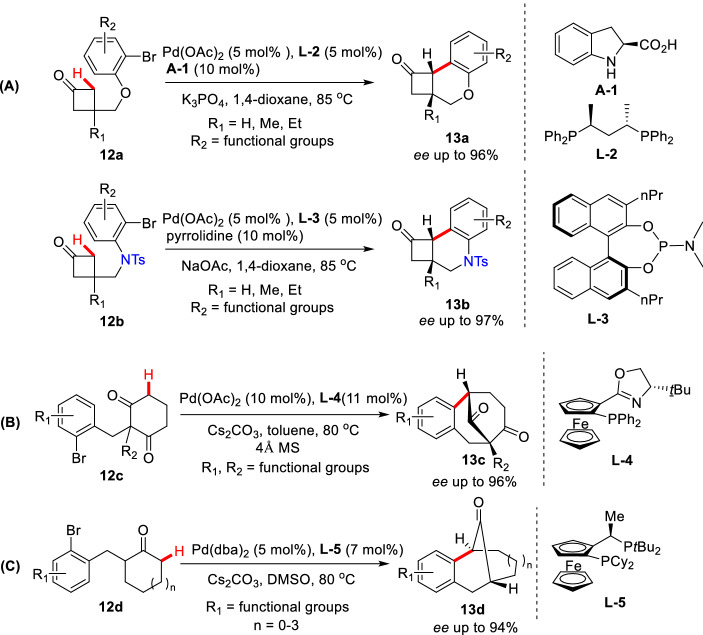
Desymmetric enantioselective *α*-C(sp^3^)-H arylations of functionalized cycloketones

Solé et al. designed nitrogen-containing aryl iodide **8a** as the substrate, and under the catalysis of PdCl_2_(PPh_3_)_2_, the intramolecular *α*-arylated product **9a** could be obtained in 92% yields, in which the pivotal active Pd complex **8aʹ** was confirmed by X-ray (Scheme 7) [31]. Jia et al. creatively combined L-proline with Pd(OAc)_2_/P(PPh)_3_/K_3_PO_4_/AcOH catalytic system to efficiently realize the intramolecular enantioselective desymmetric *α*-arylations of functionalized cyclohexanones (**10a-10j**). More importantly, this method exhibited well functional groups (FGs) tolerance and well compatibility in protonic solvent. However, the yields and *ee* values dramatically decreased when *N* was protected with acyl groups (Scheme 8) [32]. Later, Lu et al. reported the enantioselective desymmetric *α*-arylations of functionalized cyclobutanones through a similar Pd/enamine co-catalytic process (Scheme 9, A) [33]. Recently, Shi et al. represented an enantioselective desymmetric *α*-arylation of 1,3-diketones which enabled the total synthesis of (−)-parvifoline (Scheme 9, B) [34]. Zhou groups described enantioselective *α*-arylations of functionalized cycloketones via dynamic kinetic resolution of *α'*-center which led to various bridged ketones (Scheme 9, C) [35].

#### Palladium-Catalyzed Intermolecular ***α***-C(sp^3^)-H Arylations of Cyclic Carbonyl Compounds

**Scheme 10:**
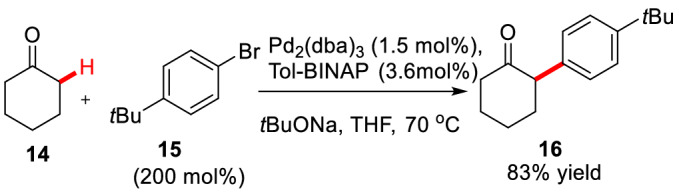
Intermolecular *α*-C(sp^3^)-H arylation of cyclohexone **14**

Buchwald et al. first reported an intermolecular Pd-catalyzed *α*-arylation of cyclohexanone **14** with 4-*tert*-butylbromobenzene **15** using Pd_2_(dba)_3_ (1.5 mol%)/Tol-BINAP (3.6 mol%) to obtain product **16** in 83% yield (Scheme 10) [36]. They speculated that the formation of active enol-palladium-aryl complex in the catalytic cycle was a key step. This speculation was later supported by the *α*-arylation of other active enol substrates [37–41].

**Scheme 11:**
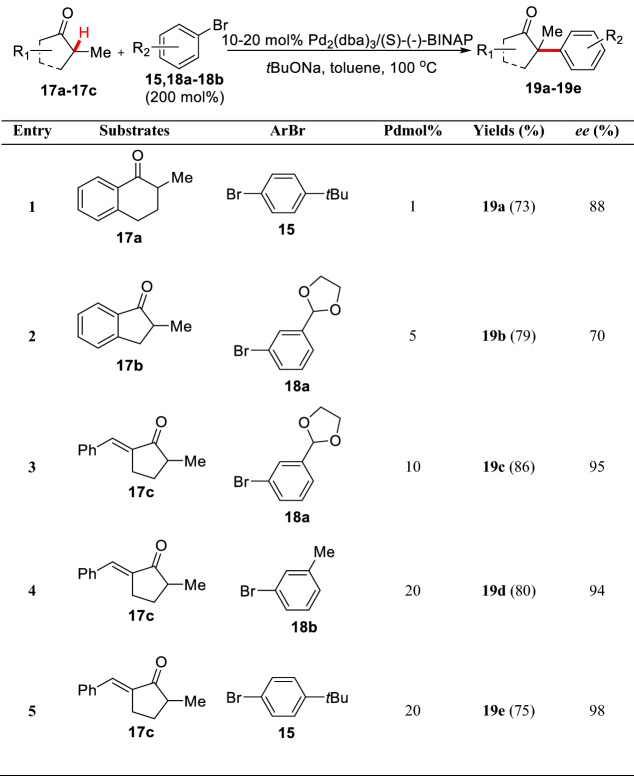
Enantioselective *α*-C(sp^3^)-H arylations of cyclic ketones reported by Buchwald et al.

**Scheme 12:**
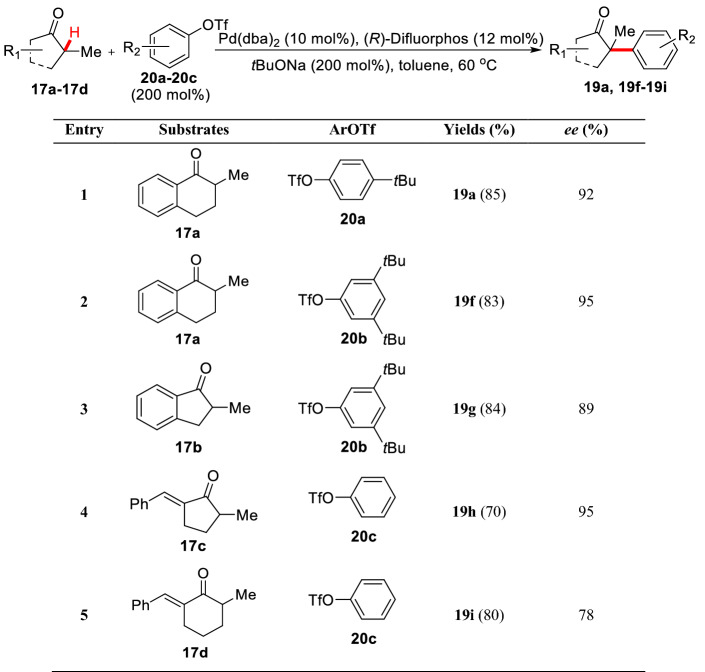
Enantioselective *α*-C(sp^3^)-H arylations of cyclic ketones reported by Hartwig et al.

Subsequently, Buchwald et al. started an asymmetric method for the first time. As a result, 2-methyl-1-benzocyclohexanone **17a** was arylated with aryl bromide **15** under the condition of Pd(OAc)_2_ (10–20 mol%)/(s)-(-)-BINAP (12–24 mol%) to give **19a** (yield 73%, 88% *ee*) [42]. The study also found that under the same catalytic system, 2-methyl-1-benzocyclopentanone **17b** was used as the substrate to react with aryl bromide **18a** leading to the arylated product **19b** with low *ee* value (70%). In view of the fact that these reactions are very sensitive to substrates and aryl bromides, Buchwald et al. screened different substrates and aryl bromides, and finally found that the products (**19c-19e**) with high *ee* value could be obtained by using cyclic ketone **17c** as substrate in cooperation with aryl bromides (**15**, **18a-18b**) (Scheme 11). They also found that the reaction could be carried out under mild conditions (50 °C) using P(*t*Bu)_3_ as the ligand in similar catalytic system [43]. Meanwhile, Hartwig et al. found enantioselective *α*-arylation of cyclic ketones (**17a-17c**) could be achieved by using phenolic trifluoromethanesulfonate (**20a-20c**) under the condition of Pd(dba)_2_/(*R*)-Difluorphos (Scheme 12). However, ketone **17d** gave the corresponding product with low *ee* value (78%) in this system [44]. Interestingly, nearly comparable yields and *ee* values were obtained by using quinine as the co-catalyst with Pd(dba)_2_ [45].

**Scheme 13:**
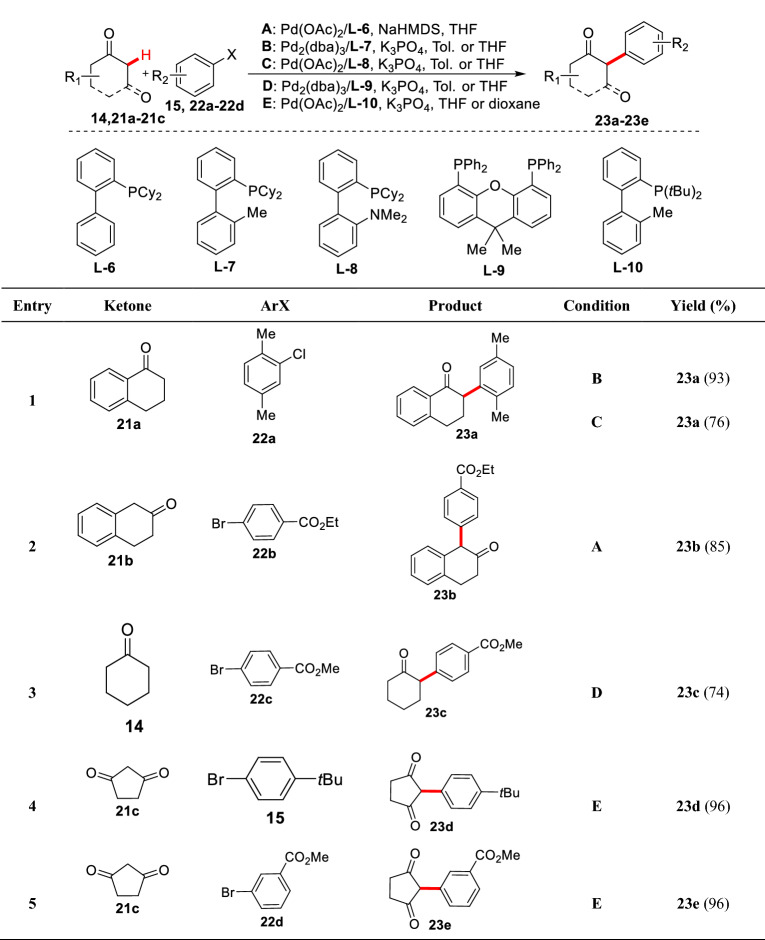
*α*-C(sp^3^)-H arylations of various cyclic ketones reported by Buchwald et al.

To further evaluate the reaction, Buchwald et al. conducted combinatorial studies on different cyclic ketones (**14** and **21a-21c**), aryl bromides (**15** and **22a-22d**), and phosphine ligands (**L-6** to **L-10**) (Scheme 13). The results showed that the large hindrance biphenyl phosphine ligands combined with Pd(OAc)_2_ or Pd_2_(dba)_3_ exhibiting high catalytic efficiency. It is worth noting that the *ortho*-substituted aryl chloride (**22a**) or *ortho*-disubstituted aryl bromide and aryl bromides containing electron-withdrawing groups (**22b-22d**) exhibited good compatibility to furnish aryl product (**23a-23e**) in 74–96% yields [46]. Similar reports showed that the *α*-C(sp^3^)-H arylations of cyclic ketones **17a** and **21a** could also be achieved by using aryl chlorides [20] and azacarbene ligands [47].

**Scheme 14:**
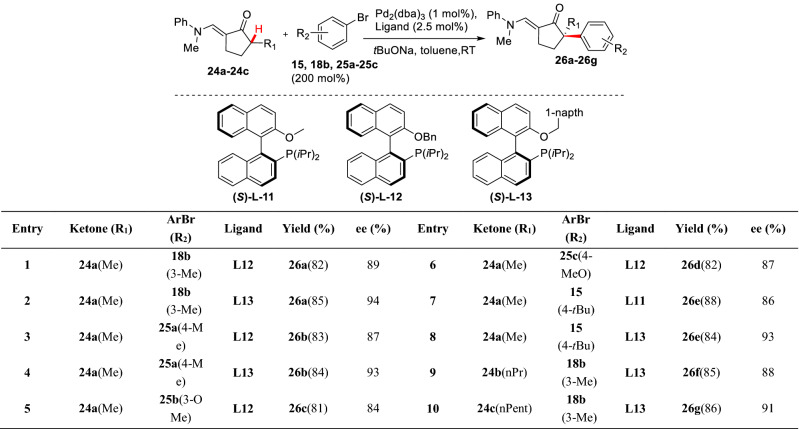
Improvements on arylation *α*-C(sp^3^)-H of cyclic ketones reported by Buchwald et al.

**Scheme 15:**
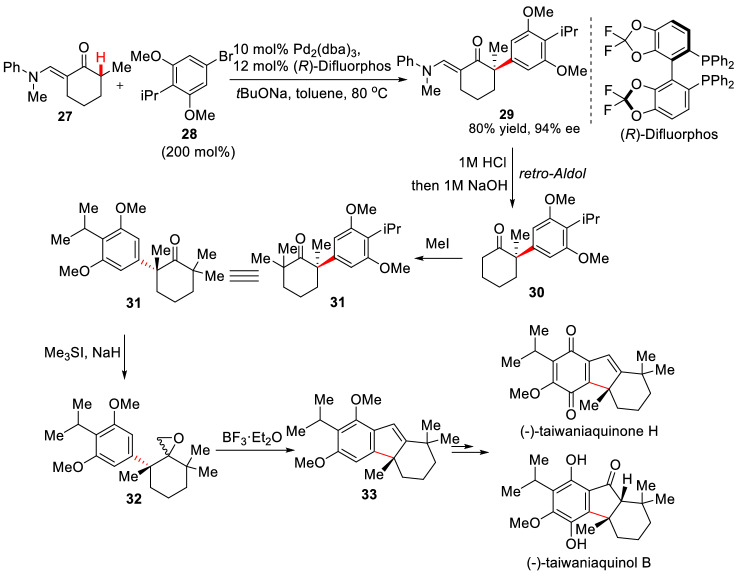
Enantioselective total synthesis of (−)-taiwaniaquinone H and (−)-taiwaniaquinol B

With those satisfactory results, Buchwald et al. continued developing such catalytic systems to finally find a new chiral catalytic coordination of phosphine ligand ((*S*)**-L-11** to **L-13**) with Pd_2_(dba)_3_. Using the system, the asymmetric *α*-arylations of substrate (**24a-24c**) were carried out at room temperature to form (**26a-26g**) (Scheme 14). Besides 4-methoxybromobenzene **25c**, 4-methyl, 4-tert-butyl, 3-methoxy, and 3-methylbromobenzene (**25a**, **15**, **25b** and **18b**) could give *α*-arylation products with high yield (72–88%) and high enantioselectivity (80–94% *ee*) [48]. Hartwig et al. reported an asymmetric *α*-arylations of ketone **27** using aryl bromide **28** (Scheme 15). In the Pd_2_(dba)_3_/(*R*)-difluorphos catalytic system, *α*-arylation product **30** was obtained with high yield (80%) and excellent enantioselectivity (94% *ee*), and the subsequent total syntheses of (−)-taiwaniaquinone H [49] and (−)-taiwaniaquinol B [50] were completed using **29** as raw materials [51] (Scheme 15).

**Scheme 16:**
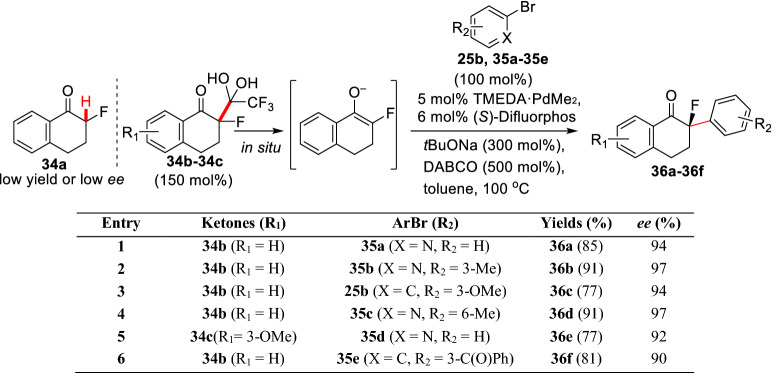
Enantioselective *α*-C(sp^3^)-H arylations of *α*-fluoro ketones reported by Hartwig et al.

In view of the fact that the *α*-substituted cyclic carbonyl compounds can form chiral fixed all-carbon quaternary stereocenter by *α*-arylation, such as compounds **19a-19i**, **26a-26g**, and **29**, which can effectively avoid the racemization of *α*-aryl compounds and subsequent reduce of optical purity. Moreover, the construction of chiral all-carbon quaternary stereocenter has always been attracted a majority of interests from the field of organic chemistry [52–58], so it is of great significance to design suitable substrates for such reactions. Hartwig et al. initiated the asymmetric *α*-arylation of *α*-fluoroketone **34a** under various catalytic systems. The results showed that all the attempts were either low yield (54%) or low ee (72%). They speculated that the enolization of **34a** was less effective when using weak base K_3_PO_4_, but *α*-fluoroketones **34b** could be completely enolized in situ by weak base [59]. Based on the systematic screening of the catalytic conditions, Hartwig et al. found that the asymmetric coupling of *α*-fluoroketones (**34b-34c**) with different aryl bromides (**25b** and **35a-35e**) could be efficiently realized under the catalysis of TMEDA·PdMe_2_/(*S*)-Difluorphosto which gave *α*-arylation product (**36a-36f**) (Scheme 16) [60].

**Scheme 17:**
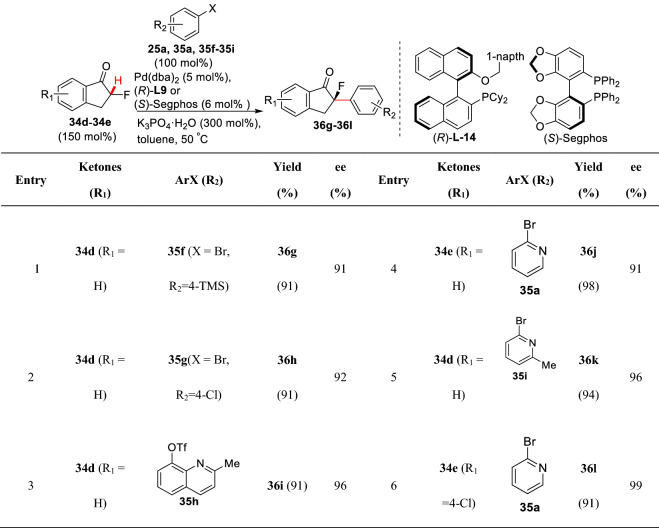
Enantioselective *α*-C(sp^3^)-H arylations of *α*-fluoro-1-indanone

As a scope extension, Hartwig et al. found that *α*-fluoro ketones **34d** and **34e** showed good compatibility. Under the catalysis of Pd(dba)_2_/(*R*)**-L-14** or (*S*)-segphos, **34d** and **34e** could react with different aryl bromides or phenolic trifluoromethanesulfonate to form *α*-arylated products (**36g-36l**) with excellent *ee* values (Scheme 17). Notably, pyridine, indole, and other nitrogen-containing aryl fragments could be well controlled [60].

**Scheme 18:**
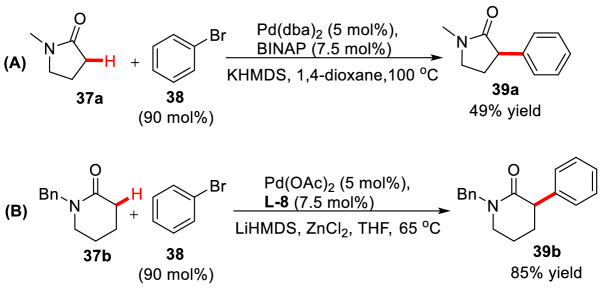
*α*-C(sp^3^)-H arylations of Lactams reported by Hartwig et al. (**A**) and Cossy et al. (**B**)

**Scheme 19:**
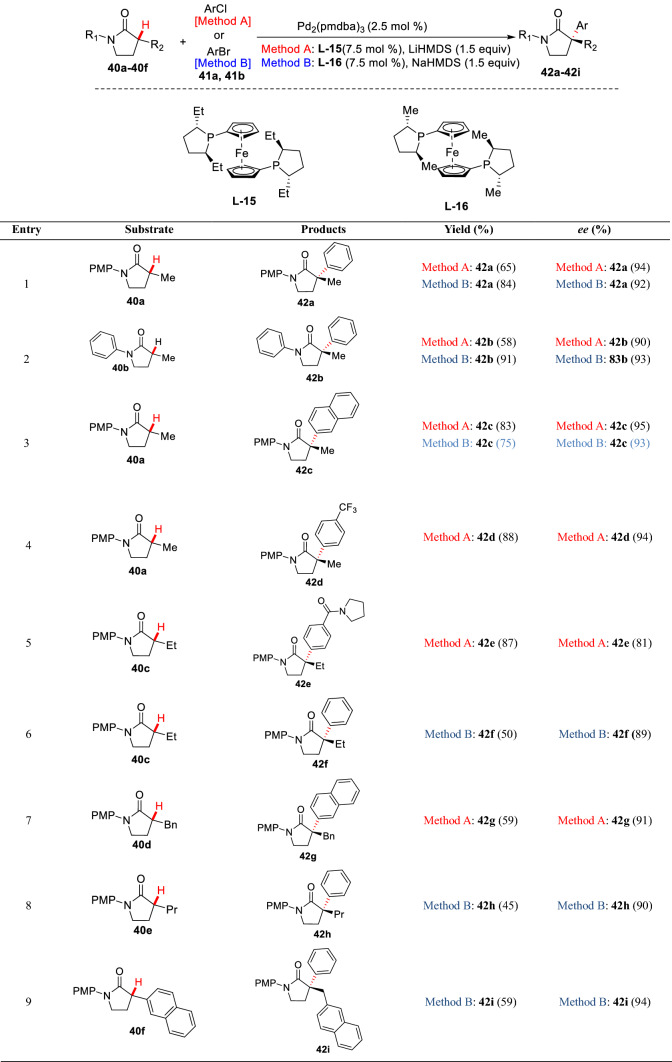
Pd-catalyzed enantioselective *α*-arylation of *γ*-lactams by Stoltz et al.

*α*-Arylation of lactam is more challenging due to its *α*-hydrogen is less acidic. In 1998, Hartwig et al. first reported palladium catalyzed *α*-arylation of *γ*-lactam **37a**. In this study, under a harsh condition of strong base (KHMDS) and high reaction temperature (100 °C), *α*-arylation product **39a** was obtained in 49% yield using Pd(dba)_2_/BINAP catalytic system (Scheme 18, A) [61]. Later, Cossy et al. described palladium catalyzed *α*-arylation of *δ*-lactam **37b** in the presence of ZnCl_2_, resulting in 85% yield of *α*-arylation product **39b** at a lower temperature (Scheme 18, B) [62]. Very recently, Stoltz et al. reported an enantioselective *α*-arylation of *γ*-lactam using Pd_2_(pmdba)_3_/**L-15** or **L-16** (Scheme 19) [63].

**Scheme 20:**
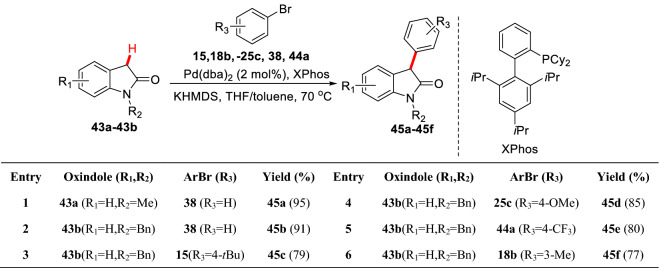
C3(sp^3^)-H arylations of *N*-substituted oxindole reported by Willis et al.

Oxindoles are a kind of small lactam molecules with important biological activities. The C3-position (*α*-position of carbonyl) aryl substituted small molecule oxindoles are promising agents for clinical use [64]. However, due to the higher pKa value of C3-hydrogen (ca. 18.5) [61], it is hard to deprotonate to form enol, which makes the arylation of C3-position more limited. Willis et al. systematically screened the C3-arylation conditions of *N*-methyl-oxindole **43a** leading to a catalytic system of Pd(dba)_2_/XPhos/KHMDS/THF/Toluene. Under this condition, *N*-substituted oxindole (**43a-43b**) were successfully arylated at C3 position with different aryl bromides (**15**, **18b**, **25c**, **38**, and **44a**) to give corresponding products (**45a-45f**) (Scheme 20) [65].

**Scheme 21:**
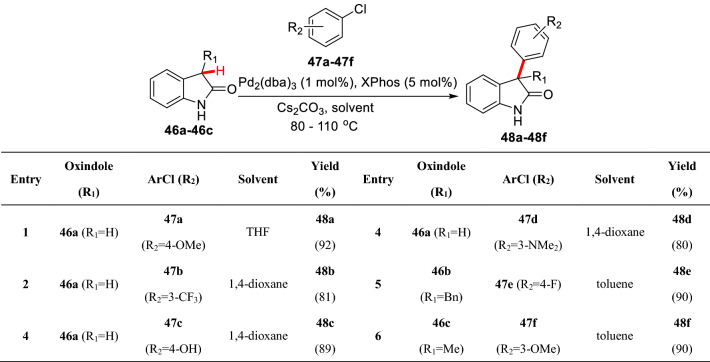
C3(sp^3^)-H arylations of *N*1 unprotected oxindole reported by Buchwald et al.

**Scheme 22:**
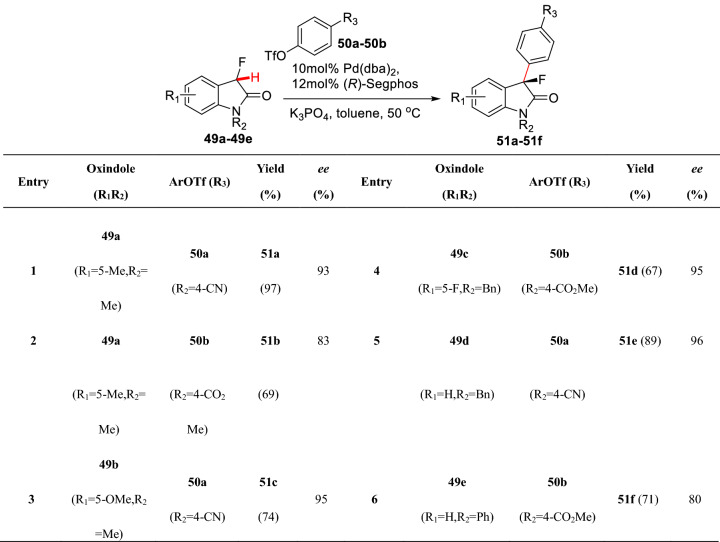
Asymmetric C3(sp^3^)-H arylations of 3-fluoroxindole reported by Hartwig et al.

Meanwhile, Buchwald et al. reported C3-arylations of *N*1 unprotected oxindoles (**46a-46c**) under a similar catalytic system using aryl chlorides (**47a-47f**) as aryl sources and Cs_2_CO_3_ as base to furnish C3 arylated products (**48a-48f**), in which (**48e-48f)** smoothly formed C3 all-carbon quaternary stereocenters (Scheme 21) [66]. Recently, Hartwig et al. reported the C3 asymmetric arylation of 3-fluoroxindoles (**49a-49e**) based on their previous studies of *α*-fluoroketones [60] using phenolic trifluoromethanesulfonate (**50a-50b**) as the aryl sources (Scheme 22) [67].

**Scheme 23:**
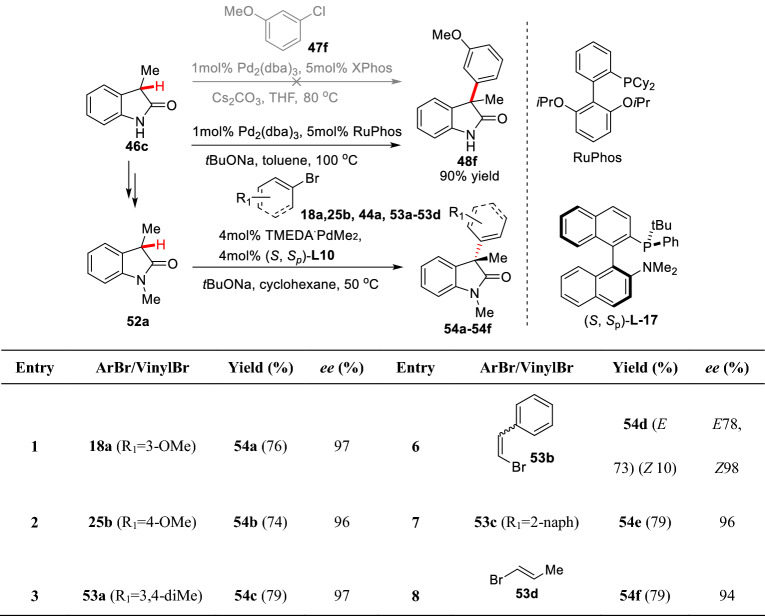
Asymmetric arylation of 1,3-dimethyl oxindole reported by Buchwald et al.

Buchwald et al. studied the asymmetric arylation of C3-methyl oxindoles. They revealed that C3-arylated product **48f** of 3-methyl oxindole **46c** could not be obtained by using aryl chloride **47f** under Pd_2_(dba)_3_/XPhos/Cs_2_CO_3_ catalytic system in THF, but could be got under the Pd_2_(dba)_3_/RuPhos/*t*BuONa system with aryl bromide **25b** in toluene (Scheme 23) [66]. Further optimization led to asymmetric arylation of 1,3-dimethyloxindole **52a** with different aryl or vinyl bromides (**44a** and **53a-53d**) in the presence of TMEDA·PdMe_2_/(*S*,*S*_P_)-**L-17** giving the C3 arylated products (**54a-54f**) with high *ee* values (94–97%) [68].

**Scheme 24:**
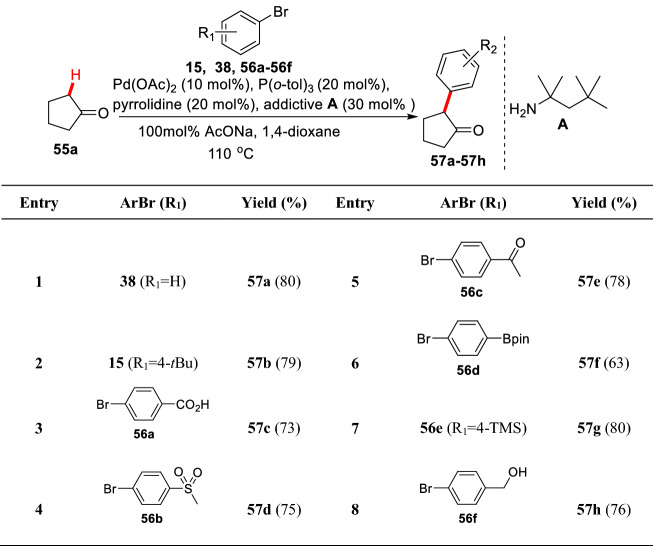
*α*-C(sp^3^)-H arylations of cyclopentanone reported by Dong et al.

Direct introduction of *N* atom into the reaction system can greatly promote the reaction by forming the Pd–N complex stabilizing the intermediate transition state and probably also activating the substrate [69–72]. Dong et al. found that palladium catalytic system could be combined with pyrrolidine to effectively achieve the *α*-arylation of cyclopentanones **55a**. In Pd(OAc)_2_/P(*o*-tol)_3_/pyrrolidine/AcONa system, some problematic aryl bromides (**56a-56f**) were well tolerable (Scheme 24) [73].

**Scheme 25:**
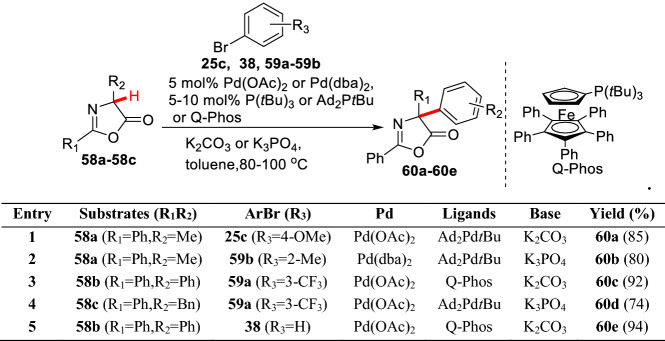
*α*-(sp^3^)-H arylations of oxazol-5-ones by Hartwig et al.

**Scheme 26:**
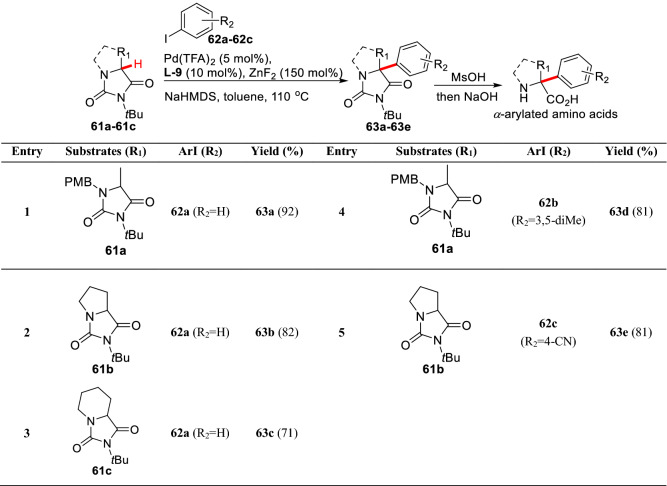
*α*-(sp^3^)-H arylations of hydantoins reported by Claden et al.

*α*-Substituted amino acids are a kind of importantly small bioactive molecules, which are fascinating tools to explore the biological process [74, 75]. Among them, the *α*-arylations of amino acids are usually prepared based on the pre-cyclic derivatives. Hartwig et al. first reported the *α*-arylation of amino acid derived lactones. It has been found that the use of bulky electro-rich ligands such as Ad_2_P*t*Bu and Q-Phos could greatly improve the reaction efficiency giving *α*-arylated products (**60a-60e**) with high yields (Scheme 25) [76]. Clayden et al. examined *α*-arylations of cyclic amino acids hydantoin derivatives (**61a-61c**) under the Pd(TFA)_2_/**L-9** catalytic system using aryl iodides (**62a-62c**) as aryl sources. They found *α*-arylated products (**63a-63e**) could be obtained in good yields through ZnF_2_-mediated transmetallation (Scheme 26) [77].

**Scheme 27:**
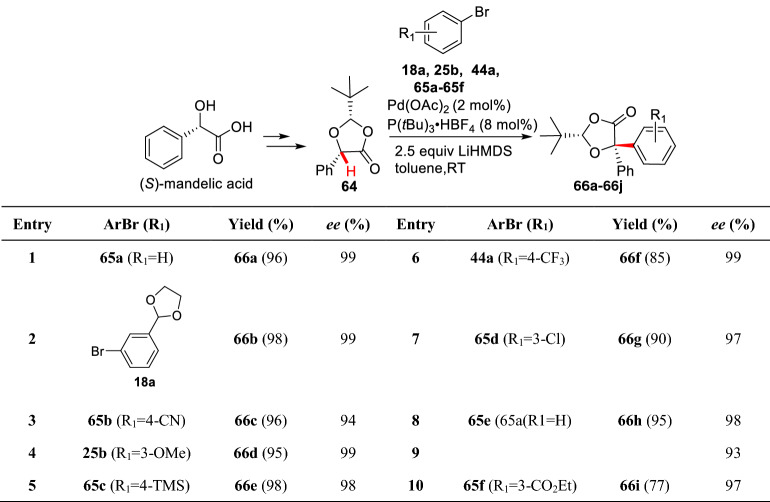
Enantioselective *α*-C(sp^3^)-H arylation of lactone reported by Jansat et al.

**Scheme 28:**
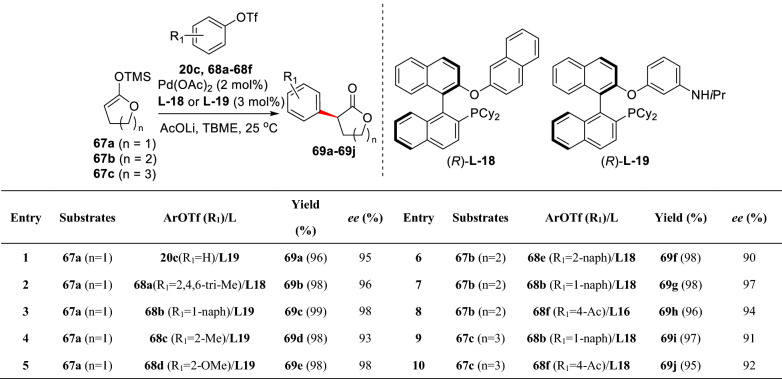
Enantioselective *α*-C(sp^3^)-H arylation of lactone-based silyl enolates by Zhou et al.

**Scheme 29:**
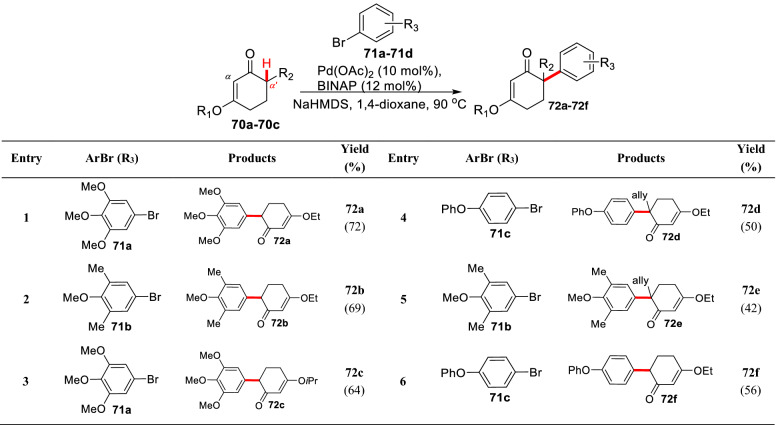
*α′*-(sp^3^)-H arylations of 3-alkoxy-2-cyclohexenones reported by Zhang et al.

Lactones are ubiquitous bioactive precursors of diols and relative analogues. Similar to lactams, conditions that involved strong base are often used to generate the enolates which may decrease the stereo-selectivity of *α*-arylation. Pd-catalyzed *α*-arylation of lactone **64** with aryl bromides (**18a**, **25b**, **44a**, and **65a-65f**) was developed by Jansat et al. using (*S*)-mandelic acid as a chiral auxiliary. In this Pd-catalyzed system, bulky P(*t*Bu)_3_ also showed a pivotal effect in both yields and stereo-selectivity, giving corresponding arylated products (**66a-66j**) (Scheme 27) [78]. Zhou et al. reported an elegant study of Pd-catalyzed enantioselective *α*-arylations of lactone silyl enolates (**67a-67c**) with phenolic trifluoromethanesulfonates (**68a-68f**) at ambient temperature. Intriguingly, some sterical hindered aryl substrates (**69b-69e**) were well comrpatible (Schemes 28 and 29) [41].

**Scheme 30:**
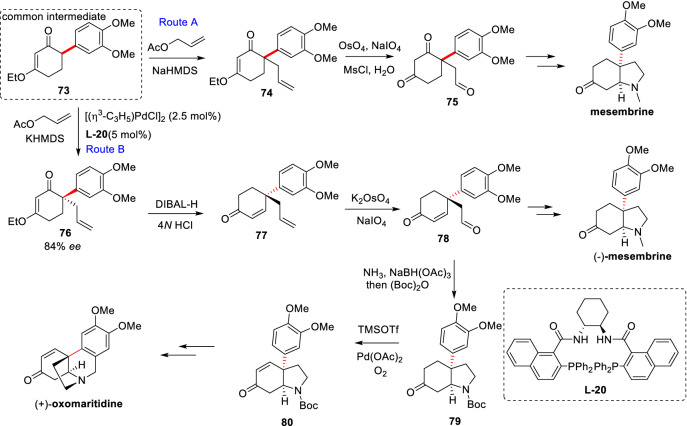
Total syntheses of mesembrine and oxomaritidine by Zhang (Route A) and Shao (Route B) et al.

Difficulties of *α′*-C(sp^3^)-H arylations of cyclic *α*, *β*-unsaturated ketones are trifold. Affected by the conjugated system, the dienolates formed from cyclic ketenes are less nucleophilic than the corresponding enolates. In which, cyclic vinylogous ester (3- or *β*-alkoxyl substituted cyclic *α*, *β*-unsaturated ketone) is more electron-rich thereby its dienolate is hardly formed by deprotonation. Furthermore, the dienolates are easy to polymerize with itself, further reducing the concentration of reaction substrate [79]. Zhang et al. first reported the *α′*-arylations of 3-alkoxy-2-cyclohexenones (**70a-70c**) using Pd(OAc)_2_/BINAP catalyst system in corporation with strong base NaHMDS (Scheme 29), which facilitated the total synthesis of the natural product mesembrine (Scheme 30, Route A) [80]. Shao et al. developed an asymmetric allylation based on common intermediate **73** further leading to the enantioselective total syntheses of (−)-mesembrine and (+)-oxomaritidine (Scheme 30, Route B) [81].

**Scheme 31:**
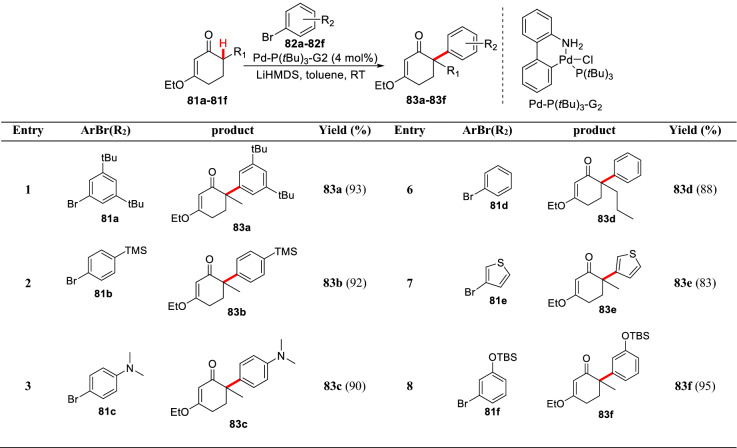
*α′*-C(sp^3^)-H arylations of 3-ethoxyl-2-cyclohexenone reported by Lautens et al.

To overcome the limitation of aryl sources in this reaction, Lautens et al. screened different palladium catalysts, and finally found that products (**83a-83f**) could be generated with high yield using the Palladium precatalyst Pd-P(tBu)_3_-G2, and *α′* arylation could be realized at room temperature (Scheme 31) [82]. The *α′*-arylation of 3-ethoxy-2-cyclohexenone can also be effectively promoted by using the bulky base [83] or phosphine ligand with large steric hindrance [84].

### Nickel-Catalyzed ***α***-C(sp^3^)-H Arylations of Cyclic Carbonyl Compounds

**Scheme 32:**
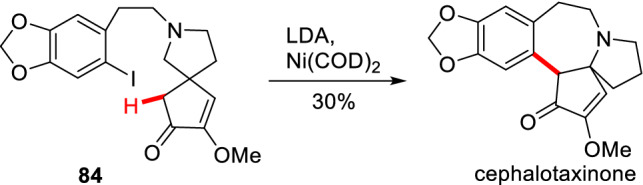
Intramolecular *α*-arylation of intermediate **84** reported by Semmelhack et al.

As a non-noble metal, nickel has also been reported to promote the *α*-aromatization of cyclic carbonyl compounds. The mechanism of Ni-catalyzed arylation of carbonyl compounds is proved to undergo a similar catalytic cycle with those of Pd-catalyzed arylation (Scheme 2) [85]. In 1973, Semmelhack et al. reported an intramolecular *α*-arylation of intermediate **84** catalyzed by Ni(COD)_2_, thus completing the total synthesis of cephalotaxinone (Scheme 32) [86].

**Scheme 33:**
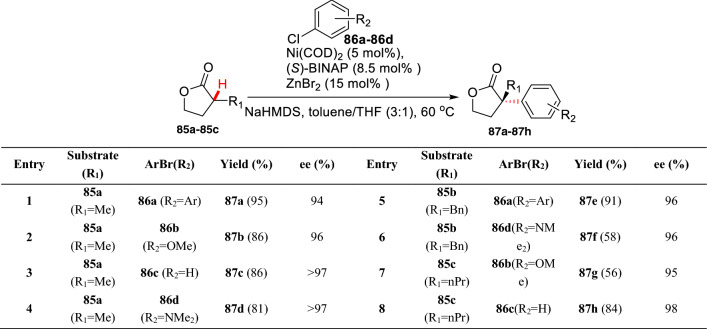
Enantioselective *α*-C(sp^3^)-H arylations of *α*-substituted *γ*-lactones

In 2002, Buchwald et al. first reported the Ni-catalyzed intermolecular *α*-arylation of *α*-methyl substituted *γ*-lactones (**85a-85c**), and the asymmetric *α*-arylation produced **87a-87h** with high *ee* values (Scheme 33) [87].

**Scheme 34:**
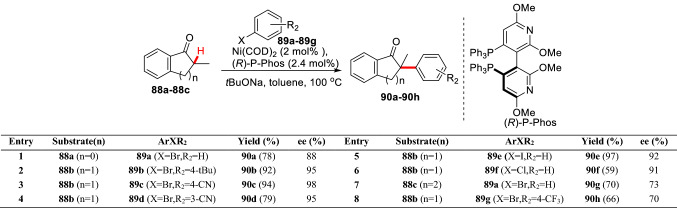
Ni-catalyzed Enantioselective *α*-C(sp^3^)-H arylation of *α*-methyl cyclic ketone

Later, Chan group reported a Ni-catalyzed asymmetric *α*-arylation of 2-methyl-2,3-dihydro-1H-inden-1-one (**88a**), 2-methyl-3,4-dihydronaphthalen-1(2H)-one (**88b**), and 6-methyl-6,7,8,9-tetrahydro-5H-benzo annulen-5-one (**88c**) with aryl bromide or iodide (**89a-89 g**). The catalytic system has good control over six-membered ring substrates, but poor control over five-membered and seven-membered ring substrates (Scheme 34) [88]. It is worth noting that a negative contribution of ZnBr_2_ to the catalytic system was observed in this work, which is contrary to the results of Buchwald et al. [87]. Hartwig et al. found that the reaction efficiency and enantioselectivity of 2-methyl-3,4-dihydronaphthalen-1(2H)-one can be significantly improved by using electron deficient phenolic trifluoromethanesulfonate as aryl source under the Ni(COD)_2_ (5 mol%)/(*R*)-Difluorophos (6 mol%) catalytic system [20]. On this basis, they further studied the relationship between the catalytic system with the aryl sources, and found that Ni(COD)_2_, (*R*)-BINAP, and benzonitrile could form the catalytic active species [(*R*)-BINAP]·Ni(*η*^2^-NCPh). Using the active species as catalyst, the *α*-arylation product of 2-methyl-3,4-dihydronaphthalen-1(2H)-one can be obtained with quantitative optical purity (*ee* > 99%) [89]. Recently, it has been reported that *α*-arylated products with better yields and higher *ee* values could also be obtained using aryl pivalate by Martin et al. [90] or indole substrate by Stanley et al. [91] under the Ni(COD)_2_/chiral ligands catalytic system.

### Other Metal Catalyzed ***α***-C(sp^3^)-H Arylations of Cyclic Carbonyl Compounds

**Scheme 35:**
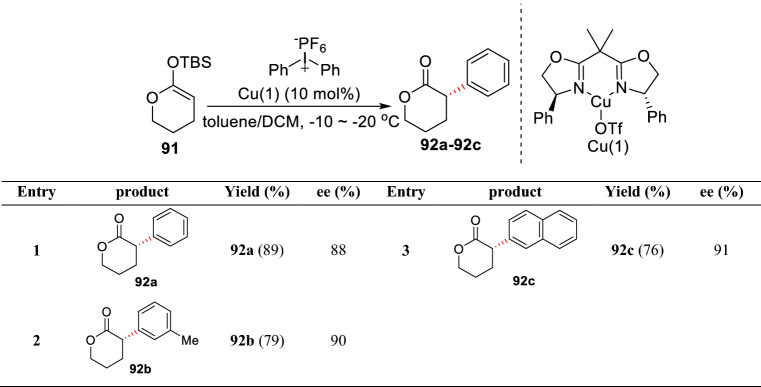
Cu-catalyzed *α′*-C(sp^3^)-H arylations of *δ*-lactone-based enol silyl ether

In 2011, MacMillan et al. reported a Cu(I)-catalyzed *α*-arylation of enol silyl ether based on *δ*-lactone **91**. The authors speculated that the highly active Cu(III) species in situ formed by the oxidation and insertion of diaryl iodonium salt promoted the reaction, which further underwent the oxidative addition and reductive elimination with enol silyl ether to furnish the final products (**92a-92c**) (Scheme 35) [92].

**Scheme 36:**
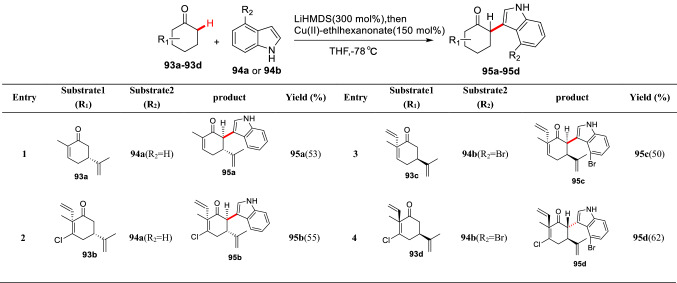
Enantioselective *α*-C(sp^3^)-H arylations of cyclic ketones reported by Baran et al.

The corresponding enolates of carbonyl compounds are easily oxidized by oxidants, such as Cu(II), Fe(III) and so on, to form the *α*-radicals, which can couple with aryl radicals to give *α*-arylated products. Baran et al. realized the oxidative coupling reaction of optically pure cyclic ketones (**93a-93d**) with indole **94a** and **94b** by using Cu(II) 2-ethylhexanoate (150 mol%), and the corresponding *α*-arylated products (**95a-95d**) were obtained in moderate yields (Scheme 36) [93–95]. With the method, they further completed the asymmetric syntheses of natural products (+)-hapalindole Q, (+)-welwitindolinone A, (+)-fischerindoles I, and (+)-ambiguine H starting from the *α*-arylated products (**95a-95d**).

**Scheme 37:**
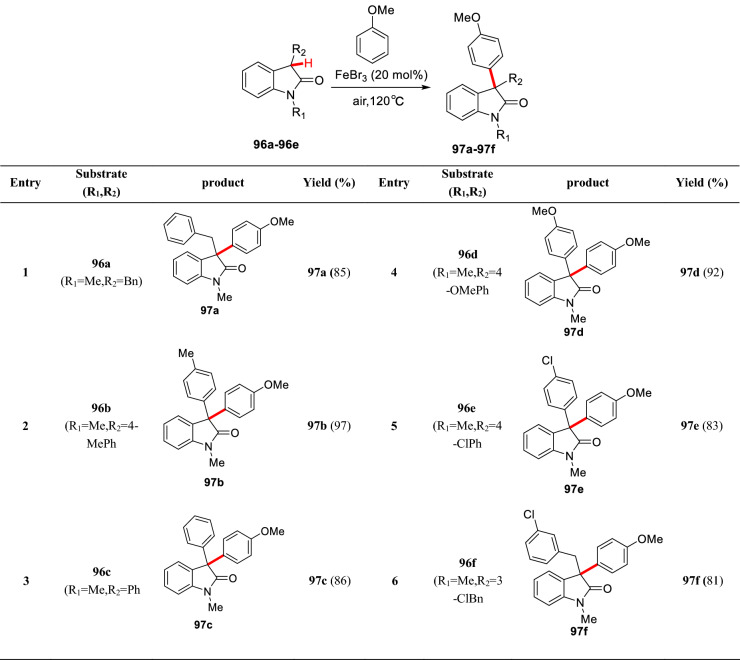
Fe-catalyzed arylation of 3-substituted oxindole reported by Li et al.

Later, Li et al. group reported a Fe(III)-catalyzed arylation of 3-substituted oxindoles (**96a-96f**). They believed that the arylation was realized through the addition of free radical in situ generated by Fe(III) or molecular oxygen at C3 position to aryl groups. Moreover, this method was well suitable for preparation of C3-arylated products with largely steric hinderance like **97a-97f** (Scheme 37) [96].

**Scheme 38:**
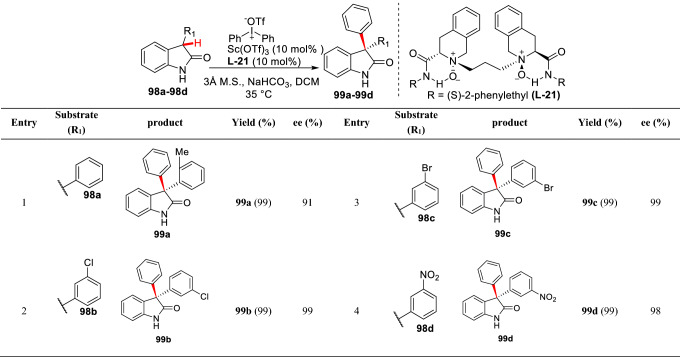
Asymmetric C3-arylations of 3-substituted oxindoles reported by Feng group

Feng et al. first described an asymmetric arylation of 3-substituted oxindole catalyzed by Sc(OTf)_3_**/L-21**. The asymmetric arylation of 3-substituted oxindoles was carried out under mild conditions, and the product **99a-99d** with excellent optical purity (*ee* > 98%) was obtained (Scheme 38) [97].

**Scheme 39:**
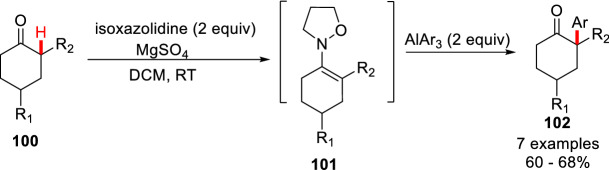
*α*-C(sp^3^)-H arylations through umpolung reaction

Umpolung reactions have been developed as unconventional methods for the synthesis of biologically active target molecules, although the umpolung arylation of cyclic enamines have been much less investigated. Miyata et al. first developed of an efficient umpolung reaction by polarity inversion at the *β*-position of *N*-alkoxyenamines **101**, which allowed *α*-arylation of various cyclic ketones **100** under mild conditions (Scheme 39) [22].

## Transition-Metal-Free ***α***-C(sp^3^)-H Arylations of Cyclic Carbonyl Compounds

**Scheme 40:**
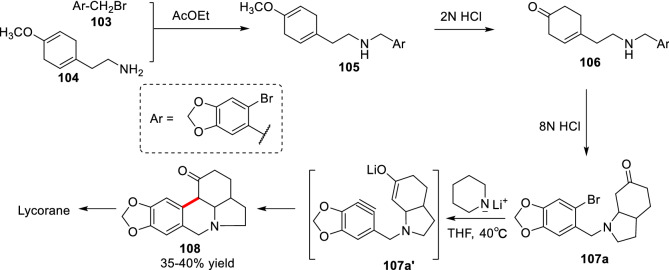
Total synthesis of lycorane via intramolecular addition of benzyne

Benzyne has high electrophilicity and is easily attacked by nucleophiles to form arylated products. As early as 1966, Ueda et al. reported the *α*-arylation through the intermediate of benzyne [98]. In the total synthesis of lycorane, they treated the intermediate **107a** with lithium piperidine to form the intermediate **107a′**, which underwent intramolecular addition to form the *α*-arylated product **108** (Scheme 40). Later, the benzyne-based arylation was applied to *α*-arylation of lactam [99], asymmetric *α*-arylation of cyclic ketone in the presence of organic small molecular amines [100]. However, the biggest problem of this method is the regioselectivity of addition to benzyne, which is difficult to apply in multiple substituted aromatic substrates.

**Scheme 41:**
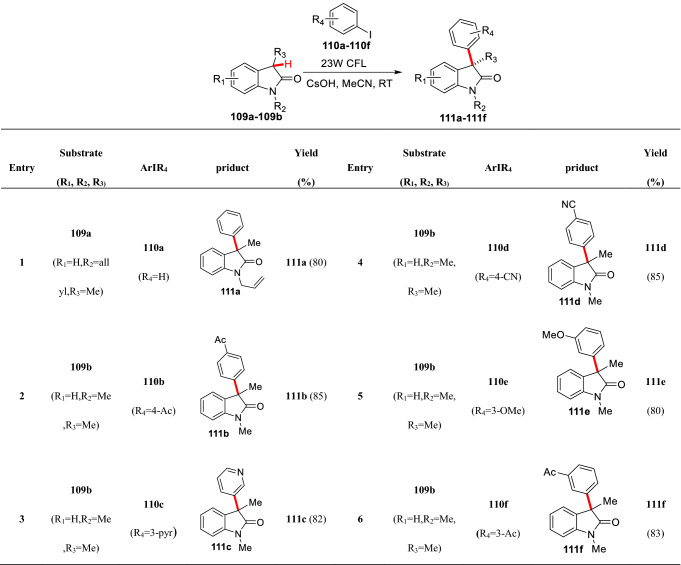
visible-light-promoted C3-arylations of 3-substituted oxindoles reported by Xia et al.

Photo-initiated S_RN_1 aromatization is an effective method for arylation of carbonyl compounds, which was first applied to the total synthesis of cephalotaxinone by semmelhack et al. [79]. Recently, Xia et al. reported the visible-light-promoted the 3-position arylation of 3-substituted oxindoles. They proved that the reaction followed the S_RN_1 pathway through the activation of aryl iodide. The method has the advantages of wide substrate compatibility (**111a-111f**), high yield (68–85%), and easy operation (Scheme 41) [101].

**Scheme 42:**
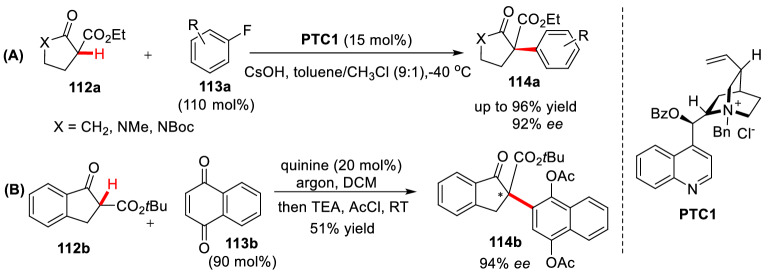
*α*-C(sp^3^)-H arylations of cyclic 1,3-diketones catalyzed by PTC (**A**) and quinine (**B**)

**Scheme 43:**
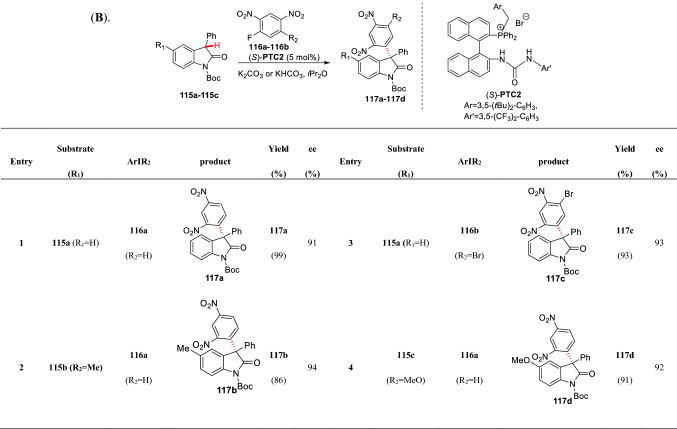
Asymmetric C3-arylations of 3-substituted oxindoles reported by Maruoka et al.

Jørgensen et al. first reported the *α*-arylation of cyclic 1,3-diketone **112a** by using phase transfer catalyst (PTC) through the nucleophilic aromatic substitution (S_N_Ar) reaction with electron deficient benzene **113a** (Scheme 42, A) [102]. Later they found quinine could also promote such transformation when using 1,4-quinone **113b** as aryl source (Scheme 42, B) [103]. Maruoka et al. successfully achieved 3-position asymmetric arylation of oxindole by the reaction of 3-substituted oxindoles (**115a-115c**) with electron deficient fluorinated aryl compounds (**116a-116c**) under the catalysis of (*S*)-PTC2 (5 mol%). The arylated products (**117a-117e**) was obtained in high yields with excellent *ee* values (Scheme 43) [104]. Subsequently, Kumar et al. found that reaction of *N*-methyloxindole and nitrobenzene in DMSO using *t*BuONa as base, the 3-arylation product could be obtained in good yields. They also proved that the reaction was realized by the addition of oxindole anion to nitrobenzene [105].

**Scheme 44:**
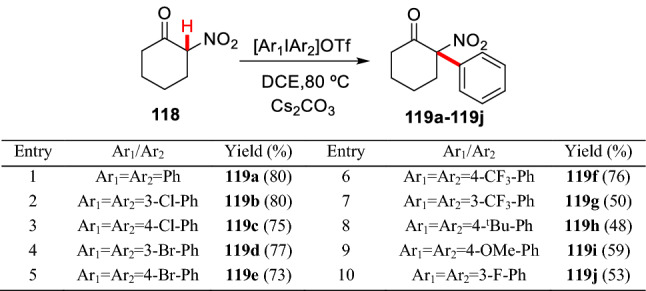
*α*-C(sp^3^)-H arylation of cyclic *α*-nitroketones with diaryl iodonium salts

Anyang et al. reported a *α*-arylation of cyclic *α*-nitroketone **118** with various diaryl iodonium salts which are environmental friendly, easy to prepare, and stable. *α*-Arylated products (**119a**-**119j**) were obtained in moderate to high yields (Scheme 44) [106].

## Conclusion

In recent years, *α*-C(sp^3^)-H arylation of cyclic carbonyl compounds has made a great progress, which provides many simple and effective methods for syntheses of molecules for biological tool, active natural products, and drugs, etc., such as 3-aryl 3-fluoroindoles, etc.. However, most of the asymmetric methods are limited to transition metal catalytic systems, problems such as how to realize green procedures and relatively small range of substrates remains to investigate. For example, Ni(0) catalyst is highly toxic, aryl halides with *ortho* hindered substituent have low compatibility in palladium catalytic system [107], asymmetric *α*-arylation of amino acids, and control of *α*-C(sp^3^)-H arylation through free radical process, etc., need to be solved.
